# Dental Treatments under the General Anesthesia in a Child with Keratitis, Ichthyosis, and Deafness Syndrome

**DOI:** 10.1155/2013/618468

**Published:** 2013-09-18

**Authors:** Sera Sımsek Derelioglu, Yücel Yılmaz, Sultan Keles

**Affiliations:** Department of Pedodontics, Faculty of Dentistry, Atatürk University, 25240 Erzurum, Turkey

## Abstract

KID syndrome is a rare genodermatosis characterized by keratitis, ichthyosis, and sensorineural deafness. Although the dermatological, ophthalmologic, and sensorineural defects are emphasized in the literature, oral and dental evaluations are so superficial. In this case report, dental and oral symptoms of a three year and five months old boy with KID syndrome, suffering severe Early Childhood Caries (s-ECC) and dental treatments done under General Anesthesia (GA) were reported.

## 1. Introduction

Keratitis-ichthyosis-deafness (KID) syndrome is congenital ectodermal disorder without a clear mode of inheritance and is characterized by erythrokeratoderma, sensorineural hearing loss, and vascularizing keratitis [1–5]. KID syndrome is usually associated with less severe keratoderma and a milder hearing problem, but the eye involvement (keratitis) may eventually lead to impaired vision [3]. Keratitis is rather a late finding and may not sometimes be seen [6]. The cause of KID syndrome was identified as a germline missense mutation in the *GJB2* (gap junction*β*-2) gene encoding for connexin-26, which is essential for gap function formation in various tissues [7, 8]. Mutations in the GJB2 gene encoding connexin 26 are detrimental to function of cochlea, palmoplantar epidermis, hair follicles, corneal epithelium, and sweat glands and ducts, causing nonsyndromic sensorineural deafness, palmoplantar keratoderma and hearing impairment, Vohwinkel syndrome, and KID syndrome [8–11].

The first component required for diagnosis is characteristic skin findings which are usually present at birth or in early infancy. The well-demarcated, erythematous, hyperkeratotic plaques with verrucous surface are distributed over face and extremities alternating with smoother areas [3]. Hyperkeratotic plaques over the face give patients an appearance of premature aging [12].

The second one is presence of sensorineural hearing loss. The third component is ophthalmologic defects, which can progress total blindness [13].

Most patients have sparse or absent scalp hair, eyebrows, and eyelashes [3]. Nails may be thickened, deformed, brittle, white, hypoplastic, or normal. In some patients teeth are normally developed, but in others they may be defective and they are likely to develop caries [3, 14–17]. However, oral mucosa, status of the primary teeth and development of permanent teeth of the children with KID syndrome, and treatment approaches for those children have not been investigated so far. Here, we report the dental restorative treatments provided for a three-year and five-month-old boy—with prediagnosed KID syndrome—under general anesthesia in a hospital setting in order to eliminate his infectious teeth and to restore his carious teeth. 

## 2. Case Report

### 2.1. Medical and Dental Histories

**Figure 1:**
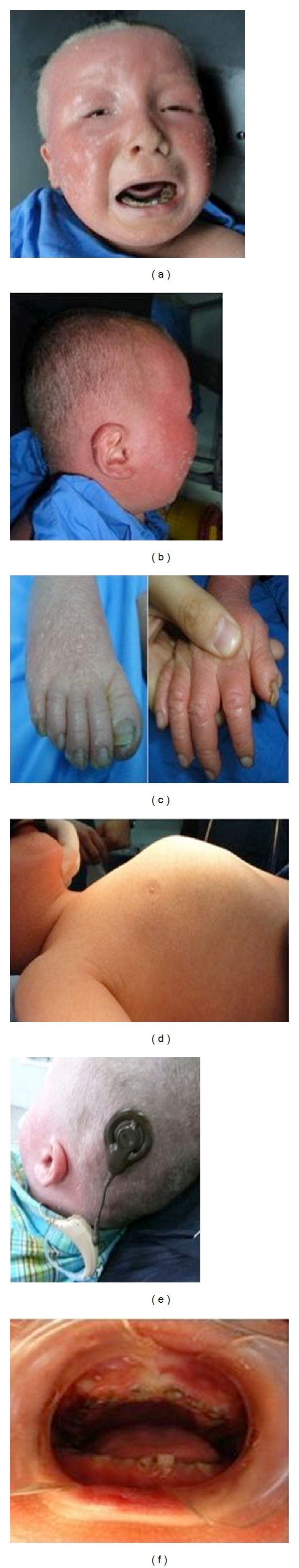
(a) Erythematous hyperkeratotic patches over the skin and the eyebrows and eyelashes are sparse in the photograph of this case. (b) Erythematous hyperkeratotic patches over the skin and the eyebrows and eyelashes are sparse in the photograph of this case. (c) View of hand, foot, and nails. (d) Pigeon chest. (e) Cochlear implant. (f) Intraoral view before the treatment.

A 3-year and 5-month-old boy was admitted to our dental clinic for treatment of his extensive carious and infectious teeth. His skin was dry with well-demarcated lesions, and there were plaques of mild hyperkeratosis on the knees and elbows. Eyebrows and eyelashes were completely absent. The hair was short, dry, and sparse. He had a cochlear implant because of sensorineural hearing loss. The patient consulted an ophthalmologist for eye functions. The ophthalmologist did not note any visual problem of the patient. The patient had a history of Atrial Septal Defect (ASD) that continued until 2 years of age and later closed spontaneously. Since the patient had a neurosensory deafness, his ability to speak was less than his peers. However, he was uncooperative, but not mentally retarded. The patient's physical appearance had the typical findings of KID syndrome except for the diagnosis of “pigeon chest (pectus carinatum),” although he had no respiratory problems due to pigeon chest (Figure 1). He can walk by himself without any assistance. None of his family members were physically and medically disabled. 

**Figure 2:**
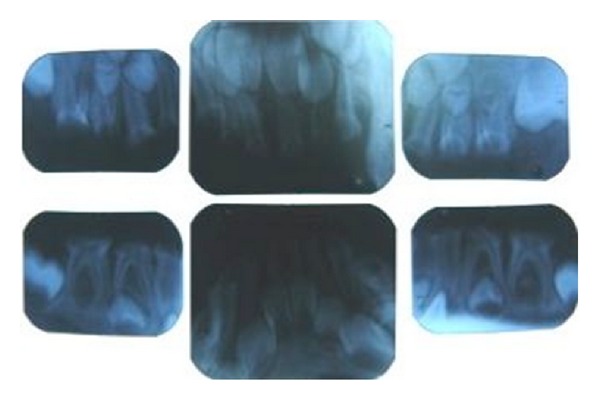
Periapical radiographs showing sEEC.

He had not received any dental treatment before. The oral mucosa was unremarkable. Intraoral examination of the child's tongue, lip mucosa, buccal mucosa, hard and soft palate, and sublingual region revealed no pathological findings. Hypoplasia, diffuse or limited opacities, fluorosis, and developmental abnormalities such as dentinogenesis imperfecta and amelogenesis imperfecta were not seen on the enamels or enamel residues. However, teeth 81, 82, 83, 84, 85, 72, 73, and 74 were vital; but with extensive caries. Teeth 51, 53, 52, 54, 55, 61, 63, 64, and 65 were nonvital because of severe caries. Two pediatric dentists diagnosed s-ECC (severe early child caries) by considering family history and dental findings (Figures 1 and 2). From ages 3 to 5, one or more cavitated, missing (due to caries), or filled smooth surfaces in primary maxillary anterior teeth or a decayed, missing, or filled score of ≥4 (age 3), ≥5 (age 4), or ≥6 (age 5) surfaces also constitutes s-ECC [18].

The child declaimed against the dental treatment and his skin was overstretched and sensitive. Also, he came from a rural area. Since the treatment could not be provided in dental office conditions, we discussed and decided that his dental treatment would be best provided under general anesthesia in a hospital setting.

### 2.2. General Anesthesia

Before each treatment session, he was referred to a cardiologist, a general pediatrician, and a radiologist in order to clear his medical status for general anesthesia. General anesthesia was induced by 2% sevoflurane using a face mask, according to a standard protocol, after an injection of the neuromuscular blocker agent, vecuronium. The anesthesia was maintained by intravenously administered propofol. Atropine and neostigmine were administered to reverse the vecuronium-induced muscle relaxation when the dental procedures were completed. The patient was intubated nasotracheally in order to obtain unobstructed surgical access into the patient's mouth, which was kept open using a molt mouth prop. A saliva ejector was used to control oral moisture, and aspiration was prevented by placing moist sterile gauze in the pharyngopalatine area. The local anesthetic agent, articaine with epinephrine, was used when oral surgery and endodontic treatment were required.

### 2.3. Dental Treatments

The aim of this dental treatment was to restore his aesthetics, speech, and chewing capacity and to eliminate the chronically infected teeth. 

The carious lesions of teeth 81, 82, 83, 72, and 73 were removed using round steel burs and the cavities were then prepared using self-etch dentin bonding agent, followed by incremental compomer resin restoration. Sof-Lex discs were used for contouring and polishing of the restored teeth. 

The pulp of the three teeth (84, 85, and 74) was removed by a spoon excavator, and bleeding was arrested with gentle pressure from a sterile cotton wool pledget (CWP) moistened with saline. A 20% ferric sulphate solution was applied to pulp stumps for 15 seconds via a CWP. After CWP was removed, zinc oxide-eugenol cement was directly placed over pulp stumps, and then the tooth was restored with high viscosity glass-ionomer cement. The prefabricated stainless steel crowns (SSC) (3M/ESPE, St. Paul, MN, USA) for pulpotomised 84, 85, and 74 teeth were cemented using luting resin-modified glass-ionomer cement.

The root canals of teeth 51, 53, 61, and 63 were filled with a calcium hydroxide + iodoform mixture paste. Then, mixture paste was removed from root canal for a distance of 2 to 3 mm. The coronal part was reconstructed by a strip crown plus compomer. 

The local anesthetic agent, articaine with epinephrine, was injected before tooth extraction. The chronically infected six teeth (52, 54, 55, 64, 65, and 75) were extracted and their alveolar sockets were sutured. 

**Figure 3:**
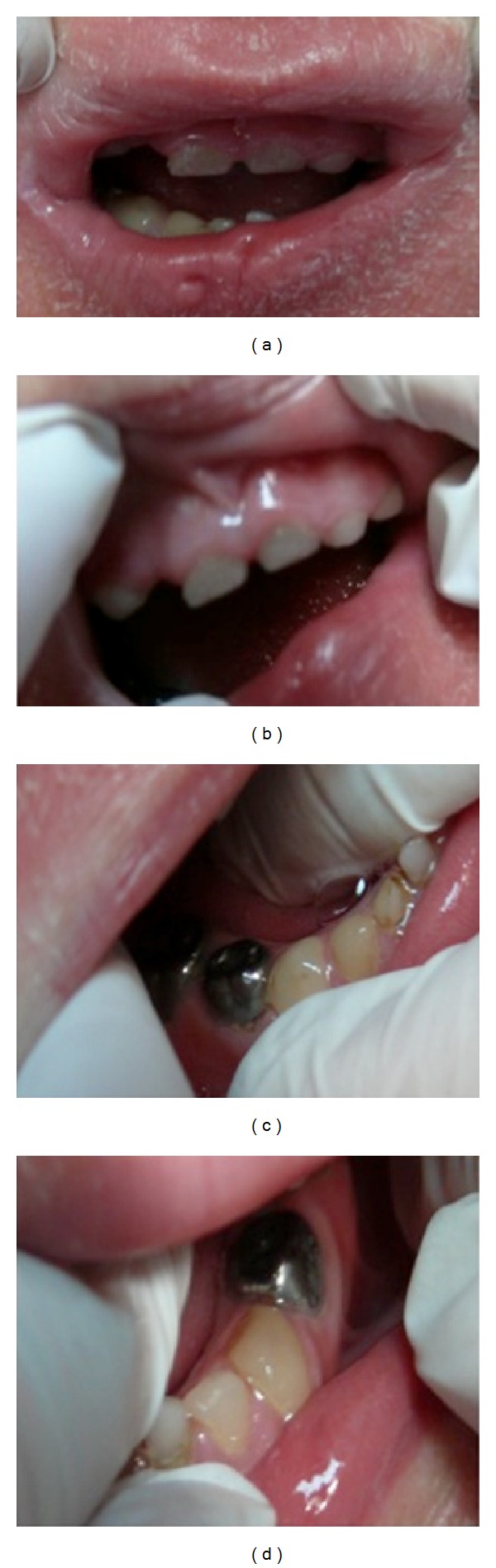
Intraoral view after the dental treatment.

After completion of the dental treatments, the patient was transferred to the recovery room, where he recovered uneventfully from the general anesthesia. All dental procedures were completed without any problems, and the entire operation took about 90 minutes. After an oral examination (Figure 3), he was discharged from the hospital the next day.

The restorations were evaluated in terms of color, aesthetics, phonetics, and parent's general satisfaction. Their scores for each evaluation criterion at each follow-up visit were ranged from excellent to good [19]. 

## 3. Discussion

The pathology which is first described by Burns in 1915 with congenital keratoderma, keratitis, and deafness findings was named KID syndrome by Skinner et al. in 1981 [1, 2]. In the world literature, nearly 100 with KID syndrome were reported [20]. Our case was identified as KID syndrome by a pediatrician and a dermatologist in 2007. Therefore, we have not signed a genetic test up again, because it would not be ethical. 

In our case, there were not only three characteristic symptoms such as ichthyosiform dermatosis, neurosensorial deafness, and but also sparse and absent eyebrow and eyelashes, nail malformations, and ASD as compatible with other case reports. The patient had a normal visual function. A vascularizing keratitis of the corneas, the third major feature of KID syndrome, occurs in about three-fourths patients. The eye symptoms usually occur by early adolescence though at some times these may appear during the fourth decade [6, 21]. The eye lesions of the KID syndrome are expressed later than the other alterations and although they are usually detected in childhood [14, 22–24], they may not evolve with symptom until puberty [25–27]. However, we had also a pigeon chest finding in our case which was not remarked in any other case reports. Pigeon chest is presented in Marfan syndrome [28], Noonan syndrome [29], Osteogenesis Imperfecta [30], Shprintzen-Goldberg syndrome [31], Loeys-Dietz syndrome [32], and Ehler-Danlos syndrome [33]. It has been specified that fatigue and asthma—ranging from mild to moderate levels—due to shortness of breath can be seen in the presence of pigeon chest [34]. In our case, we were not informed about such a symptom during the consultation before general anesthesia.

Our patient received his cochlear implant in 2009 due to hearing disability. His speaking ability was less than his peers and he had just started to make sentences. We decided to treat the child under general anesthesia because of his uncooperative manner that originated from his younger age and his communication problems. In our decision, we also considered the fact that travelling would be a problem for the far living family since the child had a lot of caries and infectious teeth should be treated for a long period of sessions. Additionally, the child's skin was so overstretched that if we chose to treat the patient with medical fixation, we could cause the skin to be cracked.

It has been stated that oral manifestations of KID syndrome might be leukokeratosis, patches of the oral mucosa, and deep fissures of the tongue or dental abnormalities [3, 15]. There were no symptoms related with tongue and mucosa. Those tissues were completely normal. Dental abnormalities in KID syndrome were mentioned in the literature [3]. However, types of the dental abnormalities were not present in the literature. We concluded that the dental situation of our patient was not the result of the KID syndrome. There was no evidence of any hereditary shape, color, or calcification disorders. Our patient was evaluated meticulously by two pediatric dentists. Two pediatric dentists decided by consensus that dental findings of the patient matched with s-ECC score for the ages over four years and his dental infections were aroused from the pulpal infections induced by the growth of the carious lesions. Our case had received no previous dental treatment. Additionally, permanent teeth were evaluated radiographically against the existence of structure and shape deformations. No structural deformation and calcification disorder were observed in permanent tooth germs. Excessive dental tissue loss or missing primary anterior teeth (space) in children may produce speech impediments of which the most familiar type is interdental (frontal) lisp, described as the inability to correctly pronounce the sounds of s, z, sh, zh, ch, and/or j, also known as the sibilant consonants [35, 36]. Children with such developmental phonetic disorders may also learn the erroneous pronunciations of those sibilants permanently and get wrong habitual tongue movements. Missing teeth in children may cause some psychological problems due to aesthetical reasons and mocking and stigmatizing of the other children because of the lips as well. Thus, in our case the nonvital upper and the lower incisors and canines were treated with root canal therapy and consequently restored with mushroom crowns. In this case, mushroom crowns are preferred because they have high clinical performance and gain utmost patient satisfaction.

 In our case, the primary molars (52, 54, 55, 64, 65, and 75) which could not be treated due to extensive chronic infection were extracted. Vital teeth with deep dentine caries were restored with SSC following the ferric sulphate pulpotomy. When restorations were evaluated in accordance with Patient Satisfaction Scale (PSS), full parent satisfaction—especially for the anterior restorations, was obtained. Removable partial denture for children for the prematurely lost primary molars was planned at a later date, when the patient grew older and became more cooperative. 

 Ventura et al. recommended that the duration of day-stay general anesthesia for a patient should be between 40 minutes and 180 minutes [37]. The duration of the dental surgery for our patient was 120 minutes and postoperation stay was two days. So the duration for the two-day stay general anesthesia was fully in compliance with the recommendation. 

## 4. Conclusion

1. Dental evaluations of children with KID syndrome and also patients' and their families' oral hygiene trainings are important for the prevention of dental problems.
2. If the children with KID syndrome had so many caries, they would have been treated under general anesthesia due to communicational and dermatological problems.
3. Supplementary dental treatments should be necessary for the patients with KID syndrome in order for the delayed speech affiliated with their hearing impediments to progress normally and also to help eliminating pronunciation disorders and psychological problems probably arising from the missing teeth.
